# High Throughput Gene Expression Measurement with Real Time PCR in a Microfluidic Dynamic Array

**DOI:** 10.1371/journal.pone.0001662

**Published:** 2008-02-27

**Authors:** Sandra L. Spurgeon, Robert C. Jones, Ramesh Ramakrishnan

**Affiliations:** Fluidigm Corporation, South San Francisco, California, United States of America; University of Cape Town, South Africa

## Abstract

We describe a high throughput gene expression platform based on microfluidic dynamic arrays. This system allows 2,304 simultaneous real time PCR gene expression measurements in a single chip, while requiring less pipetting than is required to set up a 96 well plate. We show that one can measure the expression of 45 different genes in 18 tissues with replicates in a single chip. The data have excellent concordance with conventional real time PCR and the microfluidic dynamic arrays show better reproducibility than commercial DNA microarrays.

## Introduction

Large scale gene expression analysis has become an essential tool for many biological and medical investigations. DNA microarrays are extremely powerful tools for such studies in that they allow one to probe virtually the entire transcriptome to give an overall picture of gene expression behavior [1]. However, their results for any given gene are often noisy or ambiguous [2]. Therefore it has become common practice to check the results of a genome-wide study with real time PCR (RT-qPCR), which has excellent sensitivity, dynamic range, and reproducibility and is widely regarded as the “gold standard” measurement. Unfortunately RT-qPCR is a low throughput technique, which limits the number genes that can be verified. Furthermore, many microarray studies are concluded by identifying a set of 20–100 genes which are the most important determinants of the phenomenon of interest [3]. Validation studies or practical medical application then require measurement of those genes on much larger sample sets than are practical with conventional microarrays. It is therefore of great interest to create new automation tools that increase the parallelism and throughput of RT-qPCR.

Microfluidic technology has found a number of applications in biological automation [4]. The ability to make arbitrary fluidic manipulations at the nanoliter scale has led to the development of a number of new tools with applications including protein crystallization [5], single cell gene expression [6], and cell culture [7]. It has been shown that one particularly useful way to apply such small plumbing is in the creation of microfluidic matrixes, or “dynamic arrays”, that let one perform all possible combinatorial assays on a set of reagents while realizing significant economies of scale in both pipetting, labor and reagent consumption [8]. Here we show that microfluidic dynamic arrays can be used to perform high throughput gene expression measurements with real time PCR. Single chips were used to measure the expression of 45 genes in 18 different adult and fetal tissues, and the results were compared both with microarray measurements and with conventional RT-qPCR.

## Results and Discussion

### Chip Design and Raw Data

**Figure 1:**
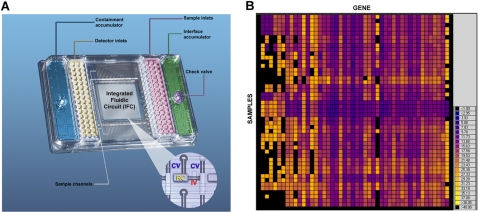
The 48.48 dynamic array chip. A. Photograph of a 48.48 dynamic array chip showing the position of the sample inlets and the detector inlets in which the gene expression assay reagents are added. The check valves allow pressure to be applied and released. The accumulators provide reservoirs to hold the pressure and keep the valves closed during the reaction. The integrated Fluidic Circuit (IFC) is in the center of the chip. This is a network of fluid lines, NanoFlex™ valves and reaction chambers. The insert shows a blow-up of a portion of the IFC with one of the 2304 individual reaction chambers (RC) and the associated containment valves (CV) and interface valve (IV) There are two containment valves and one interface valve associated with each reaction chamber. B. A computer generated image (heat map) of a 48.48 dynamic array chip obtained after thermal cycling of the chip. Each of the squares represents 1 reaction chamber from the chip. The color indicates the C_T_ value according to the legend shown on the right. Black chambers indicate a C_T_>40.

Figure 1A shows a picture of the microfluidic chip used in this study, a 48.48 dynamic array. The chip is mounted on a plastic carrier with interface and containment accumulators and 48 sample inlets and detector inlets. The dimensions of the inlets and the size of the plate conform to SBS standards [9] and are robotically compatible. The integrated fluidic circuit (IFC) is a network of fluid lines, NanoFlex™ valves and chambers. The NanoFlex™ valves are made of an elastomeric material which deflects under pressure to create a tight seal and are used to regulate the flow of liquids in the IFC. Prior to loading, the chip is primed using the NanoFlex™ IFC controller which pressurizes the control lines and closes the interface valves. Individual samples are pipetted into the sample inlets and the gene expression assays are pipetted into the detector inlets. The chip is then placed back on the NanoFlex™ IFC controller for loading and mixing. During this process, pressure is applied to the fluid in the sample inlets and the fluid is pushed into the fluid lines and then into the individual wells. At the same time the fluid in the detector inlets is pushed into the fluid lines. Mixing of the two fluids is prevented by the closed interface valve. The containment valves are then closed and the interface valves opened which pushes the reagents in the detector inlets into the individual reaction chambers to allow for mixing. At the end of the mixing the interface valves are again closed and the chip is ready for cycling. This process takes approximately 55 minutes. While some details of this chip and process have been previously described [10], [11], more details on the design, function and characterization of the dynamic array chip are in preparation (Unger M, et al. The Dynamic Array: High Throughput Real-Time Quantitative PCR by Microfluidic Large Scale Integration, manuscript in preparation, 2008).

After loading and mixing is complete, the chip is placed into the instrument for thermal cycling. Once the cycling program has started, the chip is imaged at the end of each cycle. When the cycling is completed, analysis software generates PCR curves for each of the 2304 wells. Figure 1B shows a computer generated image of the data obtained from one of the chips used for the gene expression analysis study. Forty-five gene expression assays and preamplified cDNA from 18 different tissues and 3 control preamplified cDNAs were included in this study. A complete list of the gene expression assays and cDNA samples can be found in Table S1. Each square of the image represents one of the 2304 wells on the chip. The different colors indicate different C_T_ values according to the color key shown on the right side of the figure. Squares that are black indicate that the C_T _is greater than 40. For the 48.48 dynamic array chip used here amplification curves are visible over 6 logs from a C_T_ value of 7.9 to 25.7 for a gene expression assay with an efficiency of 89% (Fig. S1, Table S2, supplemental data), which shows data across a 10-fold serial dilution across 288 replicates of the same gene. The quality of data is very high; for example, for the highest concentration (Curve set 1, relative concentration 1×10^0^), the mean Ct value was 7.9, and the standard deviation across the 288 Cts was only 0.089. This is extremely high quality data. We routinely test our chips by using a similar concentration of sample across the entire chip (2304 replicates), and typically obtain a standard deviation of about 0.1 across 2304 replicates (data not shown).The last curve has a C_T_ of 25.7; the mean copy number for that sample was measured by microfluidic digital PCR and found to be 8.

### Validation of Gene Expression Data

We compared a subset of the gene expression data obtained here to both microarray data reported for the same tissues and genes and to conventional real time PCR on microliter volume samples. GeneNote is a publicly available data set of gene expression in normal human tissues [12]. The data can be viewed at http://bioinfo2.weizmann.ac.il/cgi-bin/genenote/home_page.pl . Data for 8 of the 18 tissues used in the gene expression study were found in the GeneNote data base. These were brain, heart, kidney, liver, lung, prostate, muscle and spleen. Data for all but *MYH1* was found in GeneNote. For each gene the data from two duplicates is reported.

**Figure 2:**
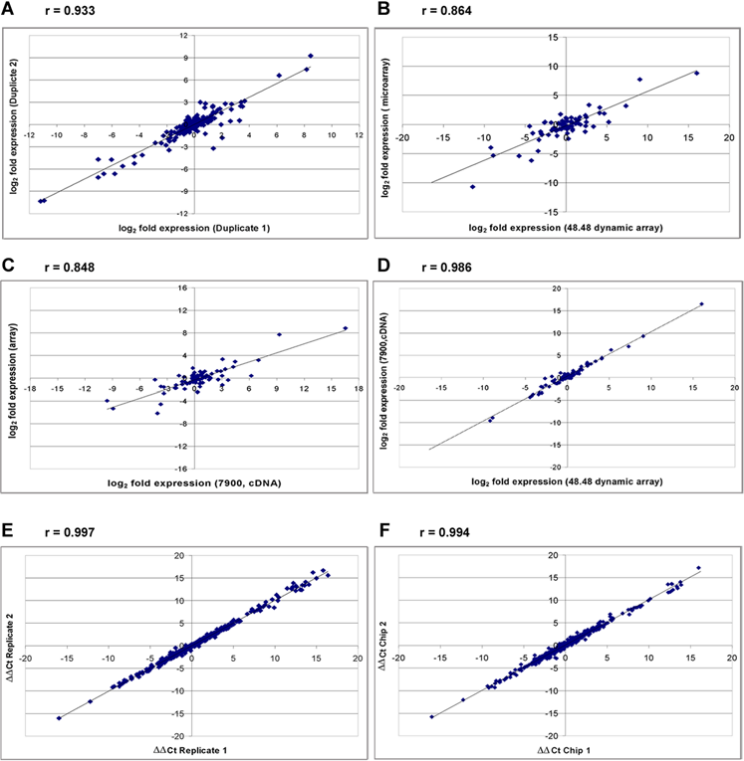
Comparisons of data. The correlation coefficient r is shown above each plot. For details regarding these comparisons see Materials and Methods. A. Comparison of Duplicate samples from the GeneNote database[10] for 45 genes and two tissues, heart and liver. B. Comparison of data from the 48.48 dynamic array obtained for preamplifid cDNA (PA cDNA) and data from the GeneNote database for the same genes and tissues. C. Comparison of the data from the 7900HT Sequence Detection System obtained with cDNA and data from the GeneNote database. D. Comparison of data from the 7900HT obtained with cDNA and the 48.48 dynamic array obtained with PA cDNA for the same genes and tissues. E. Comparison of the ΔΔC_T_ values for replicates on one of the 48.48 dynamic array chips used for gene expression analysis for all 44 genes and 18 tissues. F. Pair-wise comparison of the mean ΔΔC_T _values from two chips for the same 44 genes and 18 tissues.

The plots shown in Fig 2 A,B, C and D represent comparison data between and within measurement platforms for two tissues and 43 genes. Fig. 2A shows that the microarray data has moderate internal consistency, with a correlation coefficient between replicates of r = 0.933 and a number of significant outlier data points. In Fig. 2B the mean of the two microarray duplicates is compared to the data from the microfluidic dynamic array obtained with preamplified cDNA (PA cDNA). For these genes and tissues the correlation coefficient is only 0.864, a value that is slightly worse than the internal consistency of the micrarray data. A similar result was obtained when conventional RT-qPCR data from microliter volume samples obtained with cDNA was compared to the microarray data (Fig. 2C). Reasons for this could include a combination of systematic differences between measurement platforms as well as differences in the source tissue or in the cDNA preparation process. However, the internal noise in the microarray data is at least as large a contribution as those factors combined. The microfluidic dynamic array data obtained with PA cDNA has a high correlation with the microliter RT-qPCR data obtained with cDNA, with a correlation coefficient r = 0.989 (Fig 2D). This is similar to the result of a comparison between microfluidic dynamic array data for PA cDNA and microliter RT-qPCR data obtained with PA cDNA (r = 0.989). The reproducibility of the microfluidic dynamic array data for replicates within a chip and between different chips is very good (r>0.99) and is shown in Fig. 2E and F. Therefore microfluidic digital PCR performed on a dynamic array is able to obtain high throughput gene expression data that is essentially identical in quality to conventional microliter RT-qPCR and of superior quality to publicly available microarray data from the same tissue type.

### Tissue specificity of gene expression

**Figure 3:**
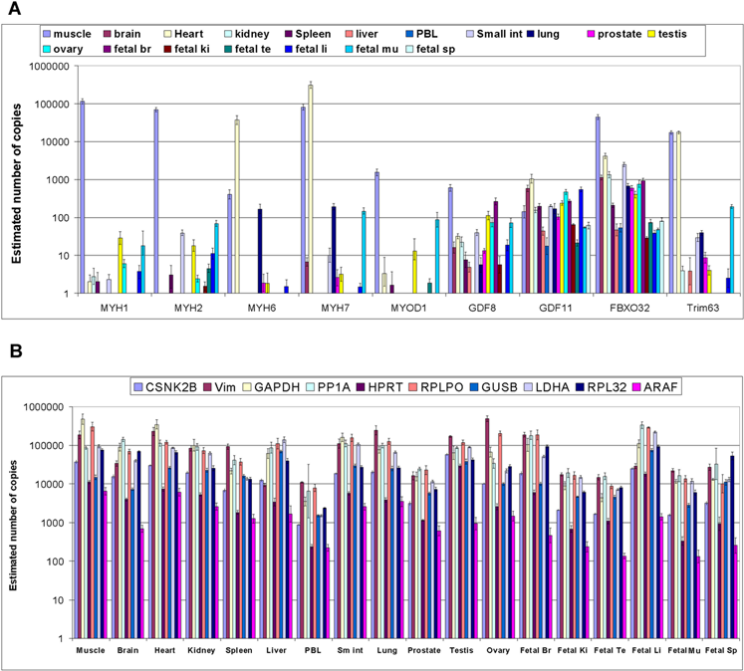
Estimated copy numbers. Using the standard curve shown in Fig. S2 the actual number of copies in the reaction chambers was estimated from the mean C_T_ values of all the data obtained on the three chips used previously. The data is plotted as the log_10_. Error bars were determined from the mean C_T_ values +/−one standard deviation. The raw data for the standard curve in Figure S2 is presented in Table S4.

We measured the expression of both muscle-specific genes and housekeeping genes. Fig. 3A shows the results from the muscle specific genes across all tissues, grouped by gene name. *MYH1(MHC IIx/d)*, *MYH2(MHC IIa)*, *MYH6(MYHCA)* and *MYH7(MYHCB)* are genes that code for isoforms of myosin heavy chain which together with myosin light chain form the subunits of myosin. Each molecule of myosin consists of two identical subunits of myosin heavy chain and two pairs of non-identical subunits of myosin light chain. Thirteen isoforms of myosin heavy chain have been identified, and their distribution shows both tissue and developmental specificity [13], [14]. The expression of these genes also varies in response to aging, exercise, and disease [15]. *MYH1* and *MYH2* are expressed primarily in adult skeletal muscle, which is consistent with the gene expression results observed here. The products of *MYH6* and *MYH7* are the predominant forms of myosin heavy chain expressed in cardiac muscle. *MYH7* is also expressed in skeletal muscle in slow twitch (Type I) muscle fibers, but the expression of *MYH6* shows a high degree of specificity for cardiac tissue. Our observations are consistent with this: the expression of *MYH6* was more than 100-fold higher in heart than muscle, and *MYH7* was similarly 4–5 fold higher. Interestingly, the absolute abundance of *MYH7* transcripts in heart was higher than *MYH6* (Fig. 3A).

The other five genes in this group are all related to muscle development and regulation of muscle mass. *MYOD1(MYOD)* codes for a myogenic regulatory factor (MRF) and is crucial for the differentiation of skeletal muscle [16]. The product of *MyoD1* is responsible for the regulation of a wide range of genes during myogenesis [17]–[19]. In the work done here expression of this gene was detected primarily in muscle and fetal muscle which is consistent with previous reports [20]. However, we also detected expression at much lower levels in heart, spleen, testis and fetal testis as shown in Fig. 3A. The proteins encoded by *GDF8*(*MSTN*) and *GDF11*(*BMP11*) are both members of the TGF-β superfamily of transcription factors [21] and are closely related proteins [22]. The product of *GDF8* is a negative regulator of skeletal muscle growth also known as myostatin and is expressed in skeletal muscle[22]. Mutations in this gene result in increased muscle mass in mice, cattle, dogs and humans [23], [24]. In the work reported here (Fig. 3A) we detected expression of *GDF8* in muscle tissue and at lower levels in other tissues as well. This result is similar to data reported for this gene in the GNF SymAtlas[25]. The protein encoded by *GDF11* is a bone morphogenic protein [26]which is involved in the regulation of anterior/posterior axial patterning in the developing embryo [27]. The expression of this gene is higher in many other tissues than in muscle as shown in Fig. 3A and the GNF SymAtlas [25]. The last two genes in this group, *FBXO32* (*ATROGIN1* or *MAFbx*) and *TRIM63*(*MURF1*) both code for E3 ubiquitin ligases and their overexpression is associated with muscle atrophy [28]. The expression of both these genes was found in muscle. Lower levels of expression of *FBXO32* were detected in all the other tissues, similar to the data reported in GeneNote [12]. The expression of *TRIM63* was detected more specifically in muscle, heart and fetal muscle than in other tissues.

In comparison to the muscle specific genes, the set of housekeeping genes is expressed across all of the tissues (fig 3B). Of the genes included here, expression of CSNK2B was the most stable across all of the tissues as determined by both geNorm [29] and Normfinder [30] and for this reason was used as the reference gene for the relative gene expression data. It is interesting to note that the relative expression levels of pairs of housekeeping genes are not necessarily constant between tissues. This illustrates one of the pitfalls of normalizing gene expression results to another gene – it is extremely challenging, if not impossible, to find genes which are expressed at constant levels across all tissues. Reliable quantitative analysis of gene expression levels requires a procedure to obtain absolute calibration, which can be accomplished either by creating defined concentrations of synthetic cDNA for each gene, or less laboriously by performing digital PCR on the original cDNA sample [6]. Digital PCR involves the isolation and amplification of single DNA molecules, which is made possible by the use of the Fluidigm digital array chip [6].

In conclusion, we have shown that the use of microfluidic dynamic array chips for real-time gene expression analysis is a rapid and reliable method for high throughput gene expression analysis. The 48.48 dynamic array chip enables 2,304 individual reactions to be analyzed with less pipetting than is required to set up of a conventional 96 well plate. The data has excellent concordance with conventional real time PCR and has better reproducibility than DNA microarrays.

## Materials and Methods

### Instrumentation and microfluidic chips

48.48 dynamic array chips and 12.765 digital array chips were from Fluidigm Corporation. The NanoFlex ™ 4-IFC Controller and the BioMark ™ Real-Time PCR System are manufactured by Fluidigm Corporation. The Νanoflex ™ 4-IFC Controller utilizes pressure to control the valves in the chips and load samples and gene expression assay reagents into the reaction chambers. The BioMark system is a real-time PCR instrument designed to thermal cycle these microfluidic chips and image the data in real time. Pre-amplification reactions were done in a GeneAmp PCR System 9700 from Applied Biosystems. Real-time PCR reactions in 384 well plates utilized an ABI Prism 7900HT Sequence Detection instrument from Applied Biosystems.

### Real-Time PCR Assays

First strand cDNA samples from 18 different human tissues were purchased from OriGene, Inc. Universal human cDNA was purchased from BioChain Institute, Inc. Taq Man Universal Master Mix, TaqMan PreAmp Master Mix and gene expression assays were from Applied Biosystems. Prior to use the 20x gene expression assays were diluted to 10x using the DA Assay Loading Reagent (Fluidigm PN 85000735). For preamplification of cDNA samples the TaqMan PreAmp Master Mix was used according to the manufacturer's directions except for the amount of the final dilution [31]. Reactions used either 6.25 µL of cDNA in a 25 µL reaction or 1.25 µL of cDNA in a 5 µL reaction and were cycled using the recommended program for 14 cycles. At the end of the cycling program the reactions were diluted 1:5. Preamplified cDNA prepared in this way was either utilized immediately or stored at −20°C until needed. Validation of the preamplification reaction with the 45 gene expression assays used in this study was done on the 7900 following the protocol as described by the manufacturer. Universal cDNA was used for this analysis and one of the genes, *CSNK2B*, was used as the reference gene. Only 3 of the 44 genes expression assays tested gave a value outside of +/−1.5 ΔΔC_T_. The values obtained with the assays for *ACTB* and *ENSA* were +3.24 and +2.82, respectively. The ΔΔC_T_ value obtained for the *GAPDH* assay was 1.52 with the universal cDNA but was below that value when tested with cDNA from four other tissues. For the assay for *CCND1* expression the ΔΔC_T_ value was +1.35 but was slightly higher when this analysis was repeated with other cDNA samples. The mean value obtained for 5 different cDNA samples was +1.63 ΔΔC_T _for the *CCND1* assay. For real-time gene expression assays on the dynamic array chips, the TaqMan Universal Master Mix was modified by the addition of 1/10 volume of DA Sample Loading Reagent (Fluidigm PN 85000735). Preamplified cDNA was added to the modified 2x TaqMan Universal Master Mix to make the final concentration of Master Mix 1.1x in the samples. Prior to loading the samples and assay reagents into the inlets, the chip was primed in the NanoFlex™ 4-IFC Controller. Five µL of sample prepared as described was then loaded into each sample inlet of the dynamic array chip and 5 µL of 10x gene expression assay mix was loaded into each detector inlet. The chip was then placed on the NanoFlex™ 4-IFC Controller for loading and mixing. After approximately 55 minutes the chip was ready for thermal cycling and detection of the reaction products on the BioMark™ Real-Time PCR System. The cycling program used consisted of 10min at 95°C followed by 40 cycles of 95°C for 15 sec and 1 min at 60°C. Data was analyzed using the BioMark Gene Expression Data Analysis software to obtain Ct values and/or ΔΔCt values. The number of replicates and the composition of the samples varied depending on the particular experiment. Details of individual experiments are given in the Results and Discussion Section. Reactions for analysis on the 7900HT Sequence Detection System consisted of 5 µL of TaqMan Universal Master Mix, 1 µL of 10x Gene Expression Assay Mix and either cDNA or preamplified cDNA for a final volume of 10 µL per well of a 384 well plate. The cycling program consisted of a 10 min incubation at 95°C followed by 40 cycles of 95°C for 15sec and 60°c for 1 min.

### 12.765 Digital array chips

For each sample 13 µL of sample reaction mix was prepared using 10 µL of TaqMan Universal Master Mix, 1 µL DA Sample Loading Reagent (Fluidigm PN 85000735) and 2 µL of the appropriate 10x gene expression assay previously modified by the addition of DA Assay Loading Reagent (Fluidigm PN 85000735). Seven µL of cDNA, preamplified cDNA or water was added to the sample reaction mix. The solution was mixed and 9.5 µL was added to each of two sample inlets. The chip was then placed on the NanoFlex™ IFC Controller and the samples were automatically loaded into the individual reaction chambers. The chip was then placed on the BioMark System for thermal cycling and detection of products. Data was analyzed using the BioMark digital array software and the numbers of positive chambers were corrected to estimate the true number of copies [32]. This number was used to determine the number of copies in the original sample.

### Determination of relative gene expression

Relative gene expression values were determined using the 2^−ΔΔCT^ method of Livak and Schmittgen [33]. *CSNK2B* was used as the reference gene and muscle was used as the reference sample. Values for ΔΔC_T_ were obtained directly from the software.

### Comparison to Microarray Data and other data comparisons

The microarray data was obtained from GeneNote [10]. The data used was MAS5.0 Normalized data. Since all of the data from the 48.48 dynamic array chips and the 7900 platform was reported as fold-expression relative to muscle, the data from the microarray for liver and heart was converted to a similar format using muscle as the reference. In cases where microarray data from more than one probe was reported, the data which matched the RT-qPCR data most closely was used. For comparison to the RT-qPCR data the mean of the two duplicates was used. All expression data has been plotted as log_2 _for comparison to RT-qPCR data. For intra- and inter-chip comparisons of 48.48 dynamic array data (Fig. 2 E and F) the ΔΔC_T_ values obtained from the software were used directly.

### Estimated number of copies in preamplified cDNA samples

Preamplified cDNA for twelve adult tissues was assayed on a 48.48 dynamic array chip with the gene expression assay for *MYH7* and a large number of replicates to determine the C_T_ values. The number of copies of *MYH7* transcript per µL of the original solution of preamplified cDNA was then determined for 10 of the samples using 12.765 digital array chips. If necessary the samples were diluted to a concentration suitable for analysis on the digital array chips. From this data the mean number of copies per chamber on the 48.48 dynamic array chip was calculated. For liver and PBL (peripheral blood leukocytes) the expression level for *MYH7* was too low to measure. The C_T_ values determined from the 48.48 dynamic array chip were plotted against the log_10_ of the concentration for samples with a mean copy number per chamber > than one. The C_T_ for single copy that was determined from the digital array data was included as the zero value on the plot. This plot was used as a standard curve to estimate the number of copies from C_T_ values for the samples used in the gene expression study.

## Supporting Information

Figure S1 A. Real-Time PCR curves for a ten-fold dilution series of preamplified cDNA generated with a gene expression assay for GAPDH. Each curve represents data from 288 individual reaction chambers. The curves for each dilution in the series agree very well except for four curves for the third dilution in the series. These four curves were localized to four adjacent chambers in on region of the chip. The most likely explanation is the presence of a flaw in this particular chip at that position. B. Plot of CT vs log10 of the dilution. Based on this curve the efficiency in this reaction is 89%. Values for the mean CT value and standard deviations of the curves are shown in Table S2.
(0.92 MB TIF)

Figure S2 Standard curve constructed from the data in Table S4 and the CT value for single copy determined from the digital array chip.
(0.18 MB TIF)

Table S1 List of Gene Expression Assays and cDNA samples. All of the assays that were used for this work are listed in the table. Assays in Groups 1 and 2 were used for the gene expression studies. The assays in Group 3 were used for the reproducibility study reported in Table S3 along with selected assays from Groups 1 and 2. The efficiency of the assays was measured in 48.48 dynamic array chips using preamplified cDNA for human muscle and with universal cDNA as template. A total of four chips were used. Error values are based on the standard deviation of 2–4 determinations. Only data that included at least five good data points was used to calculate the final number. The cDNAs were purchased from OriGene Technologies (Rockville, MD).
(0.04 MB DOC)

Table S2 Mean CT values and standard deviation for curves shown in Fig. S1. The data in this table shows the relationship between the relative concentration and CT values for GAPDH. The standard deviation is signifcantly higher for curve 6 which has a CT of 25.7. This CT value represents a mean of 8 copies per chamber as determined by analysis on a 12.765 digital array chip.
(0.02 MB DOC)

Table S3 Reproducibility of 48.48 dynamic array data. For this study the samples were preamplified cDNA from 12 normal, adult tissues. Four replicates of each sample were loaded into the sample inlets. Forty-eight gene expression assays were used which included fifteen assays for genes related to the immune and inflammatory response(Group3, Table S1) as well as an additional 33 from Groups 1 and 2 in Table S1. The mean of the CT values obtained for each of for four replicates for each of the 12 tissues was determined for all 48 assays on each Chip. The data obtained from each chip was compared in a pairwise fashion to the data from all of the other chips in a scatterplot. A total of 6 chips were run. The values determined for the correlation coefficient r and the slope are shown for all of these comparisons.
(0.02 MB DOC)

Table S4 Relationship of CT and number of copies for MYH7. Forty-seven detector inlets were loaded with the assay for MYH7 and 4 sample inlets were loaded for each sample on a 48.48 dynamic array chip. The samples contained preamplified cDNA from 12 normal, human tissues. The mean CT value and standard deviation was determined for the data from all of the positive chambers. The copies per µL was measured for each of the samples using the 12.765 digital array chip and the mean copies per 10 nL chamber was calculated from that value.
(0.02 MB DOC)
