# Supplementary figures and images for: High Throughput Gene Expression Measurement with Real Time PCR in a Microfluidic Dynamic Array
Source: PLoS One. 2008 Feb 27;3(2):e1662. doi: 10.1371/journal.pone.0001662 (PMC2244704; doi:10.1371/journal.pone.0001662)

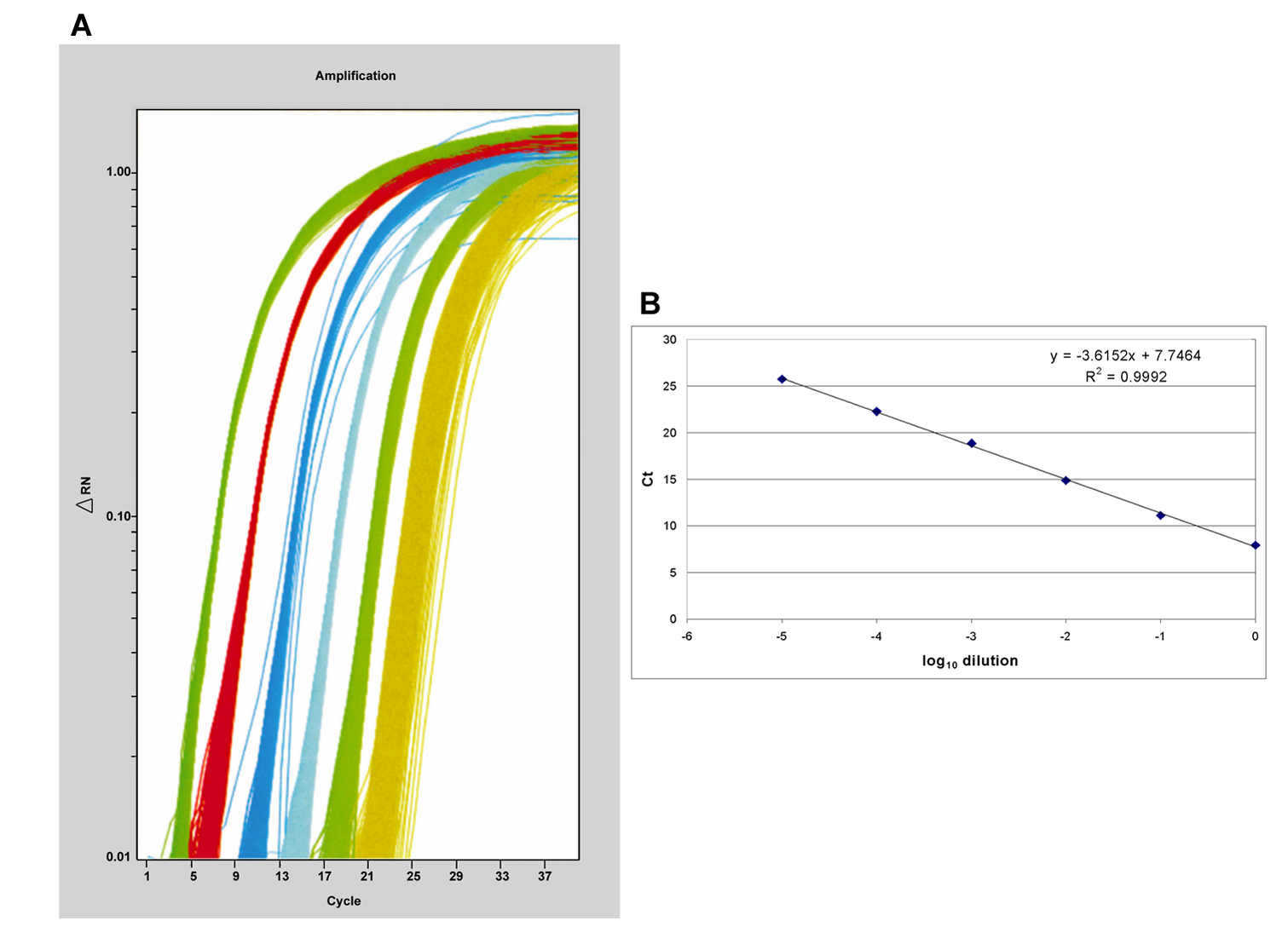

Supplement: Figure S1 — A. Real-Time PCR curves for a ten-fold dilution series of preamplified cDNA generated with a gene expression assay for GAPDH. Each curve represents data from 288 individual reaction chambers. The curves for each dilution in the series agree very well except for four curves for the third dilution in the series. These four curves were localized to four adjacent chambers in on region of the chip. The most likely explanation is the presence of a flaw in this particular chip at that position. B. Plot of CT vs log10 of the dilution. Based on this curve the efficiency in this reaction is 89%. Values for the mean CT value and standard deviations of the curves are shown in Table S2. (0.92 MB TIF) [file pone.0001662.s001.tif]

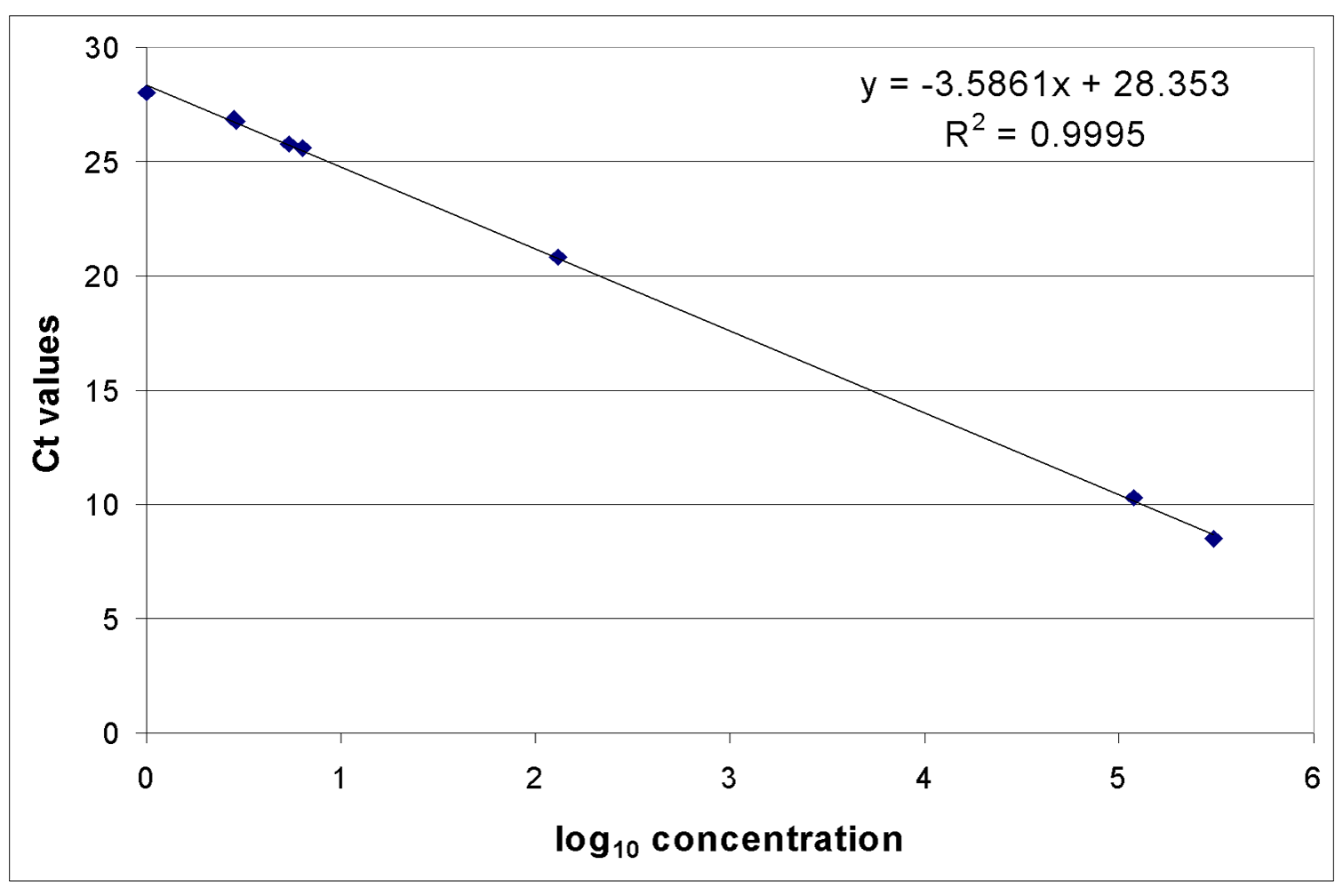

Supplement: Figure S2 — Standard curve constructed from the data in Table S4 and the CT value for single copy determined from the digital array chip. (0.18 MB TIF) [file pone.0001662.s002.tif]
