# Supplementary material for: High Throughput Gene Expression Measurement with Real Time PCR in a Microfluidic Dynamic Array
Source: PLoS One. 2008 Feb 27;3(2):e1662. doi: 10.1371/journal.pone.0001662 (PMC2244704; doi:10.1371/journal.pone.0001662)
Supplement: Table S1 — List of Gene Expression Assays and cDNA samples. All of the assays that were used for this work are listed in the table. Assays in Groups 1 and 2 were used for the gene expression studies. The assays in Group 3 were used for the reproducibility study reported in Table S3 along with selected assays from Groups 1 and 2. The efficiency of the assays was measured in 48.48 dynamic array chips using preamplified cDNA for human muscle and with universal cDNA as template. A total of four chips were used. Error values are based on the standard deviation of 2–4 determinations. Only data that included at least five good data points was used to calculate the final number. The cDNAs were purchased from OriGene Technologies (Rockville, MD). (0.04 MB DOC) [file pone.0001662.s003.doc]

Part I Gene Expression Assays

Gene Applied Biosystems Description Efficiency

Part No. %

(Group 1)

*MYH1* Hs00428600_m1 Myosin, heavy, polypeptide 1(MYHCIIx, TypeIIx) 101 +/- 8

*MYH2* Hs00430042_m1 Myosin, heavy, polypeptide 2(MYHCIIa, TypeIIa) 91 +/-11

*MYH6* Hs00411908_m1 Myosin, heavy, polypeptide 6(cardiac muscle alpha) 101 +/- 5

*MYH7* Hs00165276_m1 Myosin, heavy, polypeptide 7(cardiac muscle beta, Type I) 107 +/- 4

*MYOD1* Hs00159528_m1 myogenic differentiation 1(MYOD) 82 +/- 8

*GDF8* Hs00193363_m1 Growth Differentiation Factor 8(Myostatin) 81 +/- 3

*GDF11* Hs00195156_m1 Growth Differentiation Factor 11(BMB11) 110 +/- 3

*FBX032* Hs00369709_m1 F-box protein 32 (atrogin-1,MAFbx) 96 +/- 2

*TRIM63* Hs00822397_m1 tripartite motif-containing 63(MuRF1) 92 +/- 4

*CA9* Hs00154208_m1 Carbonic anhydrase IX nd

*CTSB* Hs00157194_m1 Cathepsin B 95 +/- 5

*EREG* Hs00914313_m1 Epiregulin nd

*FOXM1* Hs00153543_m1 Forkhead box M1 101 +/- 2

*KRT18* Hs01920599_gH Keratin 18 102 +/- 2

*PREP* Hs00267576_m1 Prolyl endopeptidase 96 +/- 4

*TIMP3* Hs00165949_m1 TIMP metallopeptidase inhibitor 3 120 +/- 1

*TNFRSF10B* Hs00366272_m1 tumor necrosis factor receptor superfamily, member 10b(DR5) 104 +/- 2

*CCND1* Hs00277039_m1 CyclinD1 (PRAD1) 96 +/- 4

*PROC* Hs00165584_m1 protein C (inactivator of coagulation factors Va and VIIIa) 83 +/- 1

*ARHGDIA* Hs00366347_g1 Rho GDP dissociation inhibitor (GDI) alpha 97 +/-2

*ATP6V1G1* Hs00606257_m1 ATPase, H+ transporting, lysosomal 13kDa, V1 subunit G1 85 +/- 2

*CD40* Hs00386848_m1 CD40 molecule, TNF receptor superfamily member 5 88 +/- 4

*ILF2* Hs00428006_g1 Interleukin enhancer binding factor 2 74 +/- 3

*CPNEI* Hs00537765_m1 Copine I 95 +/- 2

*ATP6AP1* Hs00184593_m1 ATPase, H+ transporting, lysosomal accessory protein 1 61 +/- 4

*ENSA* Hs00705265_s1 Endosulfine alpha 71 +/- 4

*IER2* Hs00270620_s1 Immediate early response 2 80 +/- 2

(Group 2)

*ACTB* (4333762F) Actin, beta 71 +/- 4

*GAPDH* (4333764F) Glyceraldehyde-3-phosphate dehydrogenase 100 +/- 2

*PPIA* (4333763F) peptidylprolyl isomerase A (Cyclophilin A) 78 +/- 6

*HPRTI* (4333768F) Hypoxantine phosphoribosyltransferase I 82 +/- 3

*PGK1* (4333765F) Phosphoglycerate kinase 1 64 +/- 6

*TFRC*  (4333770F) transferrin receptor 70 +/- 3

*GUSB*  (4333767F) glucuronidase, beta 106 +/- 2

*ALDOA* Hs00605108_g1 aldolase A, fructose- bisphosphate 96 +/- 2

*VIM* Hs00185584_m1 Vimentin 104 +/- 5

*CSNK2B* Hs00365835_m1 casein kinase 2, beta polypeptide 103 +/- 2

*RPLPO* (4333761F) ribosomal protein, large, PO 76 +/- 4

*B2M* (4333766F) beta-2-microglobulin 88 +/- 5

*CTNNB1* Hs00170025_m1 catenin (cadherin-associated protein) beta 1, 88kDa 74 +/- 4

*LDHA* Hs00855332_g1 lactate dehydrogenase A 82 +/- 2

*NONO* Hs00819149_g1 non-POU domain containing, octamer-binding 63 +/- 3

*RPL32* Hs00851655_g1 ribosomal protein L32 83 +/- 4

*USP11* Hs00234450_m1 ubiquitin specific peptidase 11 91 +/- 5

*ARAF1* Hs00176427_m1 v-raf murine sarcoma 3611 viral oncogene homolog 84 +/- 4

(Group3)

*PTGS2* Hs00153133_m1 Prostaglandin-endoperoxide synthase 2(prostaglandin G/H synthase 41

and cyclooxygenase)

*HMOX1* Hs00157965_m1 Heme oxygenase (decycling)1 89 +/- 4

*IL1R1* Hs00168392_m1 Interleukin 1 receptor, type 1 75 +/- <1

*CSF2* Hs00171266_m1 Colony stimulating factor 2 (granulocyte-macrophage) nd

*TIMP1* Hs00171558_m1 TIMP metallopeptidase inhibitor 1 99 +/- 4

*IL1A* Hs00174092_m1 Interleukin 1, alpha 37

*IL1B* Hs00174097_m1 Interleukin 1, beta 92 +/- 5

*IL2* Hs00174114_m1 Interleukin 2 nd

*CD19* Hs00174333_m1 CD19 molecule nd

*CTLA4* Hs00175480_m1 Cytotoxic T-lymphocyte-associated protein 4 92

*GZMB* Hs00188051_m1 granzyme B (granzyme 2, cytotoxic T-lymphocyte-associated nd

serine esterase 1)

*CD86* Hs00199349_m1 CD86 molecule 79 +/- 2

*ICAM1* Hs00164932_m1 intercellular adhesion molecule 1 (CD54), human rhinovirus 90 +/- 4

receptor)

*IL1RN* Hs00277299_m1 interleukin 1 receptor antagonist 74 +/- 2

*TGFB1* Hs99999918_m1 transforming growth factor, beta 1 82 +/- 8

Part II. Human cDNA samples

OriGene Part No. Tissue

CH-1001 Brain CH-1007 Small Intestine CH-1015 Fetal Brain

CH-1002 Heart CH-1008 Muscle CH-1016 Fetal Kidney

CH-1003 Kidney CH-1009 Lung CH-1017 Fetal Testis

CH-1004 Spleen CH-1010 Prostate CH-1018 Fetal Liver

CH-1005 Liver CH-1011 Testis CH-1019 Fetal Muscle

CH-1006 PBL CH-1012 Ovary CH-1020 Fetal Spleen
