# Supplementary material for: High Throughput Gene Expression Measurement with Real Time PCR in a Microfluidic Dynamic Array
Source: PLoS One. 2008 Feb 27;3(2):e1662. doi: 10.1371/journal.pone.0001662 (PMC2244704; doi:10.1371/journal.pone.0001662)
Supplement: Table S2 — Mean CT values and standard deviation for curves shown in Fig. S1. The data in this table shows the relationship between the relative concentration and CT values for GAPDH. The standard deviation is signifcantly higher for curve 6 which has a CT of 25.7. This CT value represents a mean of 8 copies per chamber as determined by analysis on a 12.765 digital array chip. (0.02 MB DOC) [file pone.0001662.s004.doc]

Relative

Curve Conc. Mean C_T_ S.D.

1 1x10^0^ 7.9 0.089

2 1x10^-1^ 11.1 0.084

3 1x10^-2^ 14.8 0.081

4 1x10^-3^ 18.8 0.107

5 1x10^-4^ 22.2 0.211

6 1x10^-5^ 25.7 0.654
