# Supplementary material for: High Throughput Gene Expression Measurement with Real Time PCR in a Microfluidic Dynamic Array
Source: PLoS One. 2008 Feb 27;3(2):e1662. doi: 10.1371/journal.pone.0001662 (PMC2244704; doi:10.1371/journal.pone.0001662)
Supplement: Table S3 — Reproducibility of 48.48 dynamic array data. For this study the samples were preamplified cDNA from 12 normal, adult tissues. Four replicates of each sample were loaded into the sample inlets. Forty-eight gene expression assays were used which included fifteen assays for genes related to the immune and inflammatory response(Group3, Table S1) as well as an additional 33 from Groups 1 and 2 in Table S1. The mean of the CT values obtained for each of for four replicates for each of the 12 tissues was determined for all 48 assays on each Chip. The data obtained from each chip was compared in a pairwise fashion to the data from all of the other chips in a scatterplot. A total of 6 chips were run. The values determined for the correlation coefficient r and the slope are shown for all of these comparisons. (0.02 MB DOC) [file pone.0001662.s005.doc]

**Chip 1 Chip2 Chip 3 Chip 4 Chip 5**

**r slope r slope r slope r slope r slope**

**Chip 1** 1 1

**Chip 2** 0.986 1.128 1 1

**Chip 3** 0.986 1.082 0.995 0.949 1 1

**Chip 4** 0.986 1.103 0.995 0.973 0.995 1.016 1 1

**Chip 5** 0.990 1.080 0.996 0.948 0.998 0.997 0.996 0.970 1 1

**Chip 6** 0.996 1.031 0.986 0.894 0.991 0.943 0.988 0.919 0.992 0.9441
