# Supplementary material for: High Throughput Gene Expression Measurement with Real Time PCR in a Microfluidic Dynamic Array
Source: PLoS One. 2008 Feb 27;3(2):e1662. doi: 10.1371/journal.pone.0001662 (PMC2244704; doi:10.1371/journal.pone.0001662)
Supplement: Table S4 — Relationship of CT and number of copies for MYH7. Forty-seven detector inlets were loaded with the assay for MYH7 and 4 sample inlets were loaded for each sample on a 48.48 dynamic array chip. The samples contained preamplified cDNA from 12 normal, human tissues. The mean CT value and standard deviation was determined for the data from all of the positive chambers. The copies per µL was measured for each of the samples using the 12.765 digital array chip and the mean copies per 10 nL chamber was calculated from that value. (0.02 MB DOC) [file pone.0001662.s006.doc]

**Tissue No.of No. of % Mean SD Copies Mean copies**

**chambers positive C_T_ per µL per chamber**

**reactions**

Brain 188 187 99.47 25.75 0.841 1504 5.4

Spleen 188 52 27.66 27.95 0.611 74 0.3

Heart 188 188 100.00 8.50 0.134 853x10^5^ 3.1x10^5^

Kidney 188 16 8.51 27.98 0.520 30 0.1

Small Intestine 188 188 100.00 25.60 0.708 1788 6.4

Prostate 188 176 93.62 26.88 1.010 781 2.8

Muscle 188 188 100.00 10.29 0.205 330x10^5^ 1.2x10^5^

Testis 188 172 91.49 26.75 0.866 807 2.9

Lung 188 188 100.00 20.81 0.195 365x10^2^ 131

Ovary 188 14 0.07 27.84 0.410 20 0.07
